# DMSO-Free Programmed Cryopreservation of Fully Dissociated and Adherent Human Induced Pluripotent Stem Cells

**DOI:** 10.4061/2011/981606

**Published:** 2011-06-01

**Authors:** Igor I. Katkov, Natalia G. Kan, Flavio Cimadamore, Brandon Nelson, Evan Y. Snyder, Alexey V. Terskikh

**Affiliations:** ^1^CELLTRONIX, San Diego, CA 92196, USA; ^2^Stem Cell Research Center, Sanford-Burnham Institute for Medical Research, La Jolla, CA 92037, USA

## Abstract

Three modes for cryopreservation (CP) of human iPSC cells have been compared: *STD*: standard CP of small clumps with 10% of CPA in cryovials, *ACC*: dissociation of the cells with Accutase and freezing in cryovials, and *PLT*: programmed freezing of adherent cells in plastic multiwell dishes in a programmable freezer using one- and multistep cooling protocols. Four CPAs were tesetd: dimethyl sulfoxide (DMSO), ethylene glycol (EG), propylene glycol (PG), and glycerol (GLY). The cells in *ACC* and *PLT* were frozen and recovered after thawing in the presence of a ROCK inhibitor Y-27632 (RI). EG was less toxic w/o CP cryopreservation than DMSO and allowed much better maintenance of pluripotency after CP than PG or GLY. The cells were cryopreserved very efficiently as adherent cultures (+RI) in plates (5-6-fold higher than STD) using EG and a 6-step freezing protocol. Recovery under these conditions is comparable or even higher than ACC+RI. *Conclusions*. Maintenance of cell-substratum adherence is a favorable environment that mitigates freezing and thawing stresses (ComfortFreeze^®^ concept developed by CELLTRONIX). CP of cells directly in plates in *ready-to-go* after thawing format for HT/HC screening can be beneficial in many SC-related scientific and commercial applications such as drug discovery and toxicity tests.

## 1. Introduction

Human pluripotent stem cells (hPSCs) hold great potential for cell therapy and regenerative medicine and as useful tools to demonstrate *in vitro* embryotoxicity. Cryopreservation (CP), storage, and shipment of hPSCs are key elements for eventual clinical applications, which will require large numbers of quality-controlled pluripotent stem cells. While effective CP protocols for ordinary cell lines and even murine embryonic stem cells (mESCs) are well established, this is not the case for hPSCs due to low recovery rates of viable and pluripotent cells following freezing, and the slow hESC growth rate, so the time from thawing to obtaining cultures suitable for experimentation can be weeks [1]. This problem is not merely an inconvenience because extended culture periods exert increased selective pressure on the cell population enhancing the likelihood of phenotypic variation and/or alterations in potency. We demonstrated [1] that standard slow freezing of hESCs with 10% DMSO causes loss of Oct4 pluripotency marker expression so that only 5–10% of the initial pluripotent cell pool remains Oct4+ after thawing [1]. To date, several attempts to optimize CP of hESCs have been made [2–16]. The effects of freezing on induced PSCs (iPSCs) are largely unexplored, except for some recent publications [17, 18]. Therefore, development and validation of efficient protocols are required in order to establish repositories for cryopreserved cells that can be thawed to yield uniform cultures. Efficient, robust, and user-friendly CP technologies for primary hPSCs would substantially shorten the time to restore cryobanked colonies, minimize potential phenotypic drift of the cells, and improve shipment safety after CP. Moreover, distribution of cells in 96-well plates could simplify end-user screening applications such as *in vitro* embryotoxicity. As such, CP remains a substantial roadblock for PSC-related applications, whether for basic research, transitional research for regenerative medicine, or development of drug screening and embryotoxicity tests where PSCs are to be used as the reporting cells [19]. 

During CP, cells undergo many steps associated with substantial stresses. The commonly used methods for CP of single cells in suspension may damage cells at several points in the process, including: (i) detachment from the dish surface, (ii) dissociation into single cells/small clusters, and (iii) centrifugation prior to aliquoting into cryovials. The cells must then be placed into a freezing medium, which usually contains one or a cocktail of several cryoprotective agents (CPAs) that can damage the cells osmotically and/or introduce specific CPA-related chemical toxicity. During cooling to suprazero temperatures, the cell membranes can undergo lipid phase transition, which if sufficiently rapid, can induce cold shock injury. During freezing, the cells can be compromised by intracellular ice if the cooling in the range between 0°C and −70°C occurs too fast or if the freezing rate is too slow, since the cells shrink as the liquid phase molality increases as the temperature drops. This excessive shrinkage is eventually lethal as a result of these solute effects. During storage, cells can experience thermal cycling due to lifting and reimmersion in the freezing cryocontainer when samples in the same container are retrieved. Cells must be thawed under optimal conditions to prevent osmotic shock, so ideally the thawing rate should match that of freezing (although for many hPSCs faster thawing rates are preferable). After thawing, the cryomedia must be removed (eluted), which may introduce additional CPA and in turn induce osmotic shock. Thawed cells are usually then replated in culturing medium and may undergo fast (e.g., necrotic) death after failure to attach, while re-attached cells may become apoptotic. 

Our goal has been to resolve the potential sources of cytotoxic stress in a systemic way. In this paper, we report first steps in this direction, namely, efficient cryopreservation of (1) dissociated, (2) adherent hPSC-derived iPSCs (3) with ethylene glycol (EG), (4) the Rho-associated kinase (ROCK) inhibitor Y-27632, and (5) CP using a programmable freezer.

## 2. General Materials and Methods

### 2.1. Derivation of hESC-iPSCs

#### 2.1.1. hESC and hiPSC Culture Conditions

Undifferentiated H9 hESCs cells and hIPSCs were cultured as described in [20]. Briefly, cells were cultured in Knockout Dulbecco's modified Eagle's medium (KODMEM, Invitrogen, no. 10829-018) supplemented with 1 mM L-glutamine with 20% Knockout Serum Replacement medium (KOSR, Invitrogen), 1 mM sodium pyruvate, 0.1 mM nonessential amino acids (NEAA, Invitrogen), 50 U/mL penicillin, 50 *μ*g/mL streptomycin (Invitrogen), 0.1 mM beta-mercaptoethanol (Invitrogen), and 8 ng/mL basic fibroblast growth factor (bFGF, Sigma no. F0291-25UG). hESCs and hiPSCs were grown on Matrigel (growth factor-reduced, BD Bioscience) coated 6-well plates (Corning, Inc. no. 3506) on a feeder layer of primary MEFs from E13.5 CD-1 mice. H9 and hiPSC lines were passaged following enzymatic digestion with collagenase IV (Invitrogen, no. 17104-019) approximately every 7 days. Cells were routinely tested for mycoplasma (MycoAlert; Cambrex, Walkersville, MD).

#### 2.1.2. Generation of H9-Derived Fibroblasts

Undifferentiated H9 cells were subjected to a standard spontaneous differentiation protocol via EB formation, as described in [20]. Briefly, collagenase IV-treated hESC colonies were dispersed by mechanical pipette trituration into cell aggregates of 500 to 800 cells. Aggregates were rinsed twice with differentiation medium (hESC medium without bFGF) before being re-plated on low attachment plates (Corning, Inc. Costar no. 3171). The medium was exchanged on day 2 and on every second day thereafter. After 6 days in suspension, EBs were transferred onto 0.1% gelatin-coated tissue culture dishes where they attached. Cells were maintained under differentiation conditions for 1–3 months and composed of heterogeneous cell populations. After 1–3 months cells were dissociated with 0.25% Trypsin-EDTA (Invitrogen) and plated onto gelatin-coated 10-cm dishes. After several passages cells had predominantly fibroblast phenotype and were named hESC-derived fibroblasts (hdFs).

#### 2.1.3. Generation of Human iPS Cells

Passages 8–10 of hdF were used for reprogramming. We applied standard Yamanaka protocol [21] with minor modifications. Original pMXs retroviral vectors encoding for *hOct4*, *hSox2*, *hKlf4,* and *hc-Myc* were obtained from Addgene and pseudotyped with VSV-G envelop protein (a gift from Gerald Pao, Salk Institute). Semiconfluent hdFs were transduced overnight with the supernatant containing all four viruses. Transduction was repeated two more times with the fresh viral supernatants. On day 6 after the first infection cells were dissociated and plated on irradiated MEFs. 5 × 10^4^ fibroblasts were seeded onto 10 cm dish. Next day medium was changed to human ES cell medium containing 8 ng/mL bFGF. Human ES cell-like colonies appeared on day 12–14 after replating on MEFs and were ready to be manually picked on day 17-18. Reprogrammed colonies were expanded and cultured as individual human iPS cell lines.

#### 2.1.4. Immunochistochemistry

The cells were rinsed with PBS, microphotographed in bright field (BF) on a CKX 41 inverted microscope, and fixed with 4% paraformaldehyde (PFA) diluted in PBS for 10 min at room temperature (RT). After permeabilization and blocking with PBSAT (3% BSA and 0.5% Triton X-100 diluted in PBS) for 1 h at RT, the cells were incubated overnight at 4°C with primary anti-Nanog mouse monoclonal antibody (Ab) (Abcam, USA) at a dilution of 1 : 200. On the following day, samples were washed with PBS and incubated for 1 hr at RT with an Alexa 555 conjugated secondary antibody (Invitrogen/Molecular Probes, USA) and counterstained with Hoechst 33342 (1 *μ*g/mL; Sigma). The cells were photographed with red and blue filters on an Olympus IX-71 inverted microscope. Anti-Oct4 antibodies were not used since they would not discriminate between endogenous and exogenous viral Oct4 expression.

## 3. Particular M & M and Results

### 3.1. Toxicity of Four Different Cryoprotectants

We earlier hypothesized that DMSO can be intrinsically toxic for hPSCs and should be substituted with a nontoxic CPA [1]. In a pilot study we found that ethylene glycol (1,2-ethane diol, EG) provided equal protection in cryopreservation of 293 cells so we considered this diol as a good candidate for substitution of DMSO. We also tested propylene glycol (1,2-propane diol, PG) and glycerol (GLY). The cells were exposed in 10% (w/w) of the CPAs for 30 min at 37°C. Recovery of the Calcein-PM+/7AAD-cell in comparison with control (QUANTA allows precise measurement of the cell concentration that was converted into total cell yield). The cells were then detached and plated according to the routine procedure, and on day 2, before the first change of the medium, the unattached cells were also harvested. The average attachment efficiency of the untreated control was in a range 65–77%, and practically 95% of the attached iPS cells were Nanog-positive after 2 days of incubation.

To exclude the osmotic damage introduced by permeable cryoprotectants, both addition and elution of the CPAs was performed in a stepwise manner according to the Fixed Shrinkage/Swelling (FSS) method developed by us previously [22]. Addition was performed in 3 steps: first, the old culture medium was aspirated, and 500 *μ*L of new medium was added. It was followed by addition of 140, 200, and 260 of the stock medium containing 2x (20%) CPA then washed twice with PBS and further incubated for 24 hrs. 

The floating clusters and single cells were then thoroughly collected by aspiration and marked as “*Non-Attached,*” centrifuged at 500 g × 10 min, washed with PBS twice, and Accutase was added to the cells for 5 min at 37°C for complete cell dissociation. The intervals for equilibration between steps were approx. 20 sec. 

Elution was performed in 5 steps: approximately 200 *μ*L of the pellet was left and 200 *μ*L, 350 *μ*L, 650, and 800 *μ*L of PBS were added with intervals approx. 20 sec. The liquid was then aspirated, and new PBS was added. 

**Figure 1:**
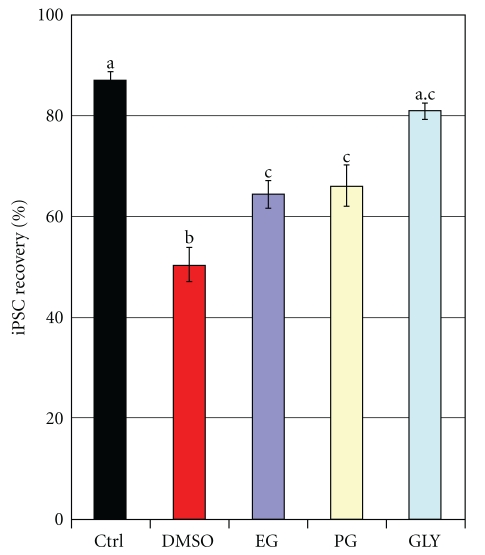
Toxicity of 4 cryoprotective agents. The cells were incubated one hour at 37°C in 10% solutions of four CPAs: dimethyl sulfoxide (DMSO), ethylene glycol (EG), propylene glycol (PG), and glycerol (GLY). Recovery of the Calcein-PM+/7AAD-cells in comparison with control (QUANTA allows precise measurement of the cell concentration that was converted into total cell yield). The cells were then detached and plated according to the routine procedure, and on day 2, before the first change of the medium, the unattached cells were also harvested. The average attachment efficiency of the untreated control was in a range 65–77%, and practically 95% of the attached iPS cells were Nanog positive after 2 days of incubation (measured by using software imaging). The actual experimental results (refered to as “*iPSC Recovery*”) normalized to the control value are depicted in Figure 3 (group “TOX”). The different letters indicate statistically significant difference among the two CPAs. *M* ± *m*, *n* = 3.

The actual experimental results (refered to as “*iPSC Recovery*”) normalized to the control value are depicted in Figure 1. We found that the toxicity DMSO was substantially higher than that of the 3 other cryoprotectants: 30-min exposure in 10%-DMSO caused loss of 30% of initially plated pluripotent cells in comparison to 5% in control untreated with the agent cells. Ethylene glycol was less toxic (76% of viable Nanog+ cell were recovered), which was comparable with PG. Glycerol was least toxic with recovery comparable with control (*P* > .1). *Results*. DMSO was the most toxic, glycerol was the least toxic, and EG and PG showed the intermediate levels of toxicity. 

**Figure 2:**
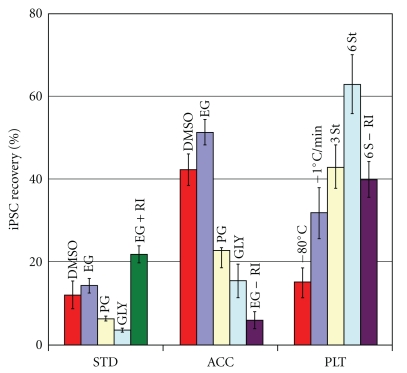
Recovery of viable iPSCs after different protocols of cryopreservation. Freezing of iPSC 3 was performed using 3 basic protocols: STD: standard routine slow uncontrolled freezing of small clumps with 10% of CPA in *Nunc* cryovials, ACC: dissociation of the cells with Accutase and freezing in cryovials and freezing and re-plating in the presence of a ROCK Inhibitor Y-27632 (RI), PLT: Freezing of adherent cells in 4-well plates in EG. Four CPAs were used in STD and ACC experiments, which are abbreviated above. The cells in ACC and PLT were frozen and recovered after thawing in the presence of RI. The numbers on the last group indicates programmed freezing protocols described in Section 3.3 and in Figure 4. Fluorescent microphotographs of some of experimental variations are shown on Figures 3 and 4. *M* ± *m*, *n* = 3.

**Figure 3:**
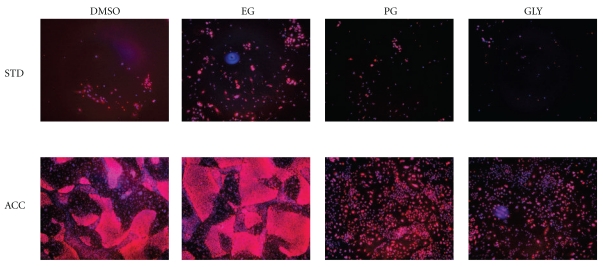
Cryopreservation of human iPSCs in vials. Fluorescent microscopy of human induced pluripotent stem cells. Color combined, Nanog (immune staining), and Hoechst. The intensity of hPGK-H2B-eGFP was weak (not shown) but quantifiable by Cell Lab QUANTA^TM^. The photos were taken on day 6 after thawing. Row STD: standard freezing with 10% of four different cryoprotective agents (CPAs) indicated in columns: dimethyl sulfoxide (DMSO) (current routine), ethylene glycol (EG), propylene glycol (PG), and glycerol (GLY). Row ACC: cryopreservation of human iPSCs after dissociation into single cells with Accutase and in the presence of ROCK inhibitor Y-27632 and different CPAs. Note: cryopreservation of dissociated by Accutase cells w/o RI resulted in 90% of cell death (Figure 2).

The toxicity of 4 CPAs can be ranked as following: DMSO > EG ~ PG > GLY (Figure 1). DMSO has been well known as a toxic agent for stem and progenitor cells, and particularly for human embryonic stem cells. It is also known as a powerful differentiation agent that may interact with the chromatin structure. We also found that DMSO is substantially more toxic to hESC than EG, PG, and GLY and comparable to ethanol and acetaldehyde (paper will be published elsewhere). In contrast, in general EG is considered less toxic in cryobiological literature, especially at high concentration used for vitrification and somewhat similar to PG, even though the data are controversial. In the majority type of cells, glycerol is considered the least toxic, and it is the least permeable. In general, a permeable CPA with smaller molecular weight such as DMSO, methanol or ethanol is the most permeable and more toxic than CPAs with higher MW such as glycerol. We have found similar effects for 2-cell mouse embryos, and for hESCs, as well as in this study. However, application of the standard freezing protocol (cells frozen in clusters in cryovials) using the same as 10% DMSO revealed that in all cases the cell recovery rate was as follows: DMSO ~ EG ≫ PG > GLY. (Figures 2 and 3, group “STD”, see Results below).

### 3.2. Cryopreservation of Dissociated iPS Cells

As described above, cell detachment from surfaces containing ECM molecules can cause massive cell death and differentiation. However, from a cryobiological perspective, CP of single cell suspensions is preferable to clusters. We address this issue by adding a Rho-kinase (ROCK) inhibitor Y-27632 (RI in the text) that helps cells withstand detachment and dissociation [32]. Addition of RI to the cell clusters also improved recovery rates but at lesser extent and was not as beneficiary in the matter of total viable yield and cell growth after thawing as for fully dissociated cells (see also our companion paper).

More striking results were obtained when detached iPSCs were dissociated into singlets using the enzyme cocktail Accutase (Figures 2 and 3, group “ACC”). In this case, although the freezing curve was not optimized (standard cooling in a Nalgene alcohol bath of −80°C was used), the recovery significantly differed from the group “STD.” Note that the absence of RI dropped the recovery dramatically. At the same time the maximum peak (EG +RI) was higher than for the STD group frozen as cell clusters.

### 3.3. Cryopreservation Attached Cells in Plates

**Figure 4:**
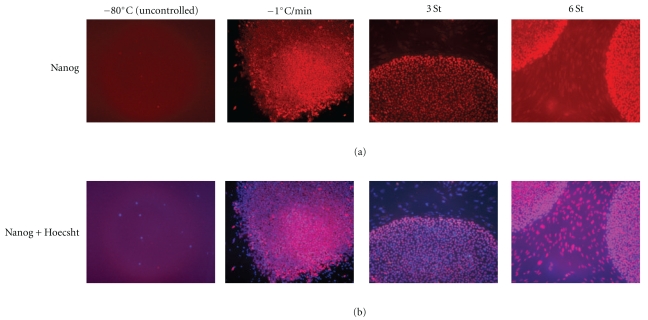
Cryopreservation of aderent human iPSCs in plates. Fluorescent microscopy of human induced pluripotent stem cells frozen in adherent state in 4-well plates. First row: Nanog (immune staining), second row: combined Nanog + Hoechst. The photos were taken on day 3 after thawing. In all cases, ethylene glycol (EG) and ROCK inhibitor Y-27632 were used.−80°C: uncontrolled freezing in a –80°C freezer in a Styrofoam box (~−1°C/min at the first stage of freezing). The yields of viable, and especially Nanog-positive cells, are very low (see Figure 3). −1°C/min: one-step freezing in a programmable freezer (Custom Biogenics, Inc, series 2100); Note 1: *eroded edges* of the colony and *differentiation* of the adjacent cells *within the colony*. 3St: three-step freezing protocol (described in Section 3.3) in a programmable freezer. 6St: six-step freezing protocol (described in Section 3.3) in a programmable freezer. Note 2: *clear distinct edges* of the colonies in BOTH cases and differentiated (*Nanog*-negative) cells (blue) *outside the colony* in the 3-Step protocol. QUANTA has confirmed substantially higher attachment efficiency and a larger 7AAD^−^/GFP^+^ pool of viable iPSCs in cases of *multistep controlled freezing* (Figure 3).

Since the standard freezing protocol requires detachment, we hypothesized that the recovery could be improved if cells were not disturbed from their “natural” environments. We thus froze cells in 4-well plates (Figure 2, group “PLT,” Figure 4). We explored several freezing protocols: (1) transferring cells into a −80°C freezer in a “Mr. Frosty” isopropanl cooler (Nalgene) for 24 hrs followed by plunging into liquid N_2_ with an initial cooling rate of ~−1°C/min; three programmed freezing protocol in a programmable freezer, namely; (2) one-step cooling from 0°C (CPA was added on ice) to −140°C at −1°C/min; (3) three- step protocol that imitates a Nalgene cryovials left at −80°C in “Mr. Frosty” ([23], Figure 6): (i) −0.63 °C/min from 0°C (CPA was added on ice) to −5°C; (ii) −0.20°C/min to −10°C; (iii) −0.48°C/min to −80°C, than transfer to LN_2_; (4) six-step programmed protocol developed by us: (i) −1°C/min from 0°C (CPA was added on ice) to −10°C; (ii) hold for 30 min at −10°C; (iii) −3°C/min to −40°C, than transfer to LN_2_; (iv) −1°C/min to −60°C; (v) −0.33°C/min to −80°C; (vi) hold at −80°C for 5 min, than transfer to LN_2_. 

*The six-step protocol yielded the best results* with 63% recovery in comparison to 10–12% for “standard” freezing. Note that when we applied the six-step approach to Accutase-dissociated cells frozen in cryovials (group “ACC”) with component X no improvement was seen apparently due to the different geometry of detached sphere-like cells and flattened attached cells. This issue needs further investigation as suspended cells may require a different freezing protocol. We are now looking to test another chemical, E, that would minimize cell damage after thawing. 

This example illustrates our approach that allowed us to obtain a sixfold increase in cell recovery (10 versus 63%) in the space of two months. Method of freezing in plates is robust and requires minimal labor. However, the best achievement required a programmable freezer, which is not expensive in comparison with the cost of additional labor and chemical required for cells frozen in one uncontrollable step (Figure 2). 

## 4. Discussion

While many aspects of these developments were used in previously documented protocols [2–16]; however, the reported results differ widely among labs and cell lines. Our clear distinction and innovation was to use the cumulated data in concert as a whole. Another problem is that a wide variety of the methods of assessment of recovery used in the abovementioned publications makes direct quantitative comparison of different results very difficult if not sometimes impossible. We have decided not to use any colony assay due to large variability of the size and amount of the colonies that can be plated into a particular well, thus, making the “*number of colonies*” as an unreliable parameter for quantification. We instead, used the number (% to the total amount of harvested cells) of GFP-positive cells that excluded a negative viability marker 7AAD as the characteristic of the attached cells. We also precisely (using QUANTA Coulter counter option) measured the amount of nonattached and attached cells because it was also impossible to plate the same amount of cells per a well even if the cells were dissociated prior to freezing and plating, and especially if they were frozen in clumps. Thus, combination of the assessment of % the cells that reattached to the surface of the well versus floating non-attached cells and the percentage of Oct4-positive viable (7AAD-) reattached cells would give us a fair assessment of the overall recovery, that is, the yield of viable pluripotent SCs after CP (*VY*). If that parameter is normalized to the yield of nonfrozen cells that are split according to the standard protocol (in clumps), that would constitute the assessment of cryosurvival (*SR*, which also includes survival to CP-related protocols such as addition and removal of the CPAs and centrifugation). Those two parameters (*VY* and *SR*) would give an objective assessment of a particular CP protocol as the whole.

We previously hypothesized that DMSO, as a potent differentiating agent, can be intrinsically toxic for hPSCs and should be substituted with a non-toxic CPA [1]. And indeed, DMSO has been found to be toxic to ESCs even in miniscule concentrations if applied for long time ([19, 24]*, our unpublished data*). In a pilot study, we found that EG provided equal to DMSO protection in cryopreservation of 293 cells [25]. We have also reported that EG was less toxic to 2-cell mouse embryos [26] while it manifested similar permeability characteristics (see [27] for references). It made EG as an attractive alternative to DMSO so we first checked the potential toxicity by exposing iPSCs to 10% v/v concentrations of DMSO, EG, and two other widely used in cryopreservation CAPs propylene glycol and glycerol. We found that the toxicity DMSO was substantially higher than that of the 3 other cryoprotectants: 30-min exposure in 10%- DMS caused loss of 30% of initially plated pluripotent cells in comparison to 5% in control untreated with the agent cells. Ethylene glycol was less toxic (76% of viable Nanog+ cell were recovered), which was comparable with PG. Glycerol was the least toxic with recovery comparable with control (*P* > .1) so the relative toxicity could be expressed as DMSO > >EG ~ PG > GLY (Figure 1). DMSO has been well known as a powerful inducer of differentiation and apoptosis [28–30].

We also found that DMSO is substantially more toxic to hESC than EG, PG, and GLY and comparable to ethanol and acetaldehyde (a paper will be published elsewhere). In contrast, in general EG is considered less toxic in cryobiological literature, especially at high concentration used for vitrification and somewhat similar to PG, even though the data are controversial. In the majority type of cells, glycerol is considered the least toxic while at the same time, it is the least permeable. In general, a permeable CPA with smaller molecular weight such as DMSO, methanol, or ethanol is the most permeable and more most toxic than CPAs with higher MW such as glycerol. We have found similar effects for 2-cell mouse embryos, and for hESCs, as well as in this study. However, application of the standard freezing protocol (cells frozen in clusters in cryovials) using the same 10% v/v concentration (though slightly different osmolality) revealed that the cell recovery rate was as follows: EG ~ DMSO > PG > >GLY. (Figures 2 and 3, group “STD”).

Cryopreservation of *dissociated hPSCs* has recently drawn attention after publications on the use of Accutase as a less harmful detaching and dissociating agent [31] and especially after Watanabe et al. reported the protective role of a Roh-associated kinas (ROCK) inhibitor Y-23672 after hES cell detachment and dissociation [32]. In all recently available reports [8, 10, 17, 18, 33–35] the authors found beneficial role of cryopreservation of hES and iPS cells after dissociation and application of Y-23672 even though direct comparison of the efficiency is not possible mostly due to different methods of estimation of viability and recovery of pluripotent cells and PS cell colonies. The mechanism of the protective role of RI can be as inhibitor of apoptosis that was caused by the activation of the ROCK pathways during dissociation and detachment, pointed out in the original Watanabe's work; at the same time, freezing (and cooling) *per se* can cause specific cold and osmotic related damages such as rearrangement of F-actin, activation of p53, and ROS production [36]. Overall, as we can see in Figure 1, CP of dissociated cells had clear advantage over the standard CP in clumps, especially if EG was used (51% versus 8% resp., *P* < .001), in a range reported by other investigators as well as in good concordance with our data on Oct4/eGFP-infected human ESCs (results will be published elsewhere). We did not explore addition of Y-23672 one hr prior freezing for CP of iPSCs as others did [10, 35] but we found it somewhat more beneficial for CP of Oct4/eGFP-infected human ESCs in comparison to addition of Y-23672 after freezing only.

Cryopreservation of adherent stem cells in monolayers in plates is a relatively novel method. Beside some anecdotic reports on freezing mouse ESC plates, and recent work by Nagy and colleagues [37, 38] and Miyamato et al. [39], very few reports are published on CP of adherent human pluripotent SCs [7, 40]. We believe that CP in adherent state is not only convenient for several applications such as high cell-based throughput and high-content screening but it also causes less damage so we (CELLTRONIX) developed in 2006–2008 a “universal” technological approach that we call ComfortFreeze^®^, that can be applied not only to the pluripotent stem cells but hESC/iPSC-derived neural and cardiac progeny as well (results to be published elsewhere).

Efficient, robust, and customer-friendly CP technologies for primary hPSCs would substantially shorten the time to restore cryobanked colonies, improve shipment safety, eliminate possible biased selective pressure within a PSC-line after CP and distribution of cells frozen in 96-well plates that could be used immediately for embryotoxicity and drug screening in PSC-based toxicology *in vitro* kits.

## 5. Conclusions

Both dissociation of iPSCs with Accutase in the presence of a ROCK inhibitor and ethylene glycol AND programmable freezing in adherent stage (ComfortFreeze^®^) have drastically improved (up to 6-fold) the yield of pluripotent cryopreserved stem cells in comparison to the standard freezing in clumps without ROCK inhibitor. Cryopreservation of adherent cells in plates has also showed a higher efficacy and lower time for restoration of the colonies to the unfrozen level than dissociation with Accutase.
